# A lifeline to treatment: the role of Indian generic manufacturers in supplying antiretroviral medicines to developing countries

**DOI:** 10.1186/1758-2652-13-35

**Published:** 2010-09-14

**Authors:** Brenda Waning, Ellen Diedrichsen, Suerie Moon

**Affiliations:** 1 Boston University School of Medicine, Department of Family Medicine, Boston, MA, USA; 2 UNITAID, Geneva, Switzerland; Utrecht University, Utrecht, Netherlands; 3 Sustainability Science Program, Center for International Development, Harvard Kennedy School of Government, Cambridge, MA, USA

## Abstract

**Background:**

Indian manufacturers of generic antiretroviral (ARV) medicines facilitated the rapid scale up of HIV/AIDS treatment in developing countries though provision of low-priced, quality-assured medicines. The legal framework in India that facilitated such production, however, is changing with implementation of the World Trade Organization Agreement on Trade-Related Aspects of Intellectual Property Rights, and intellectual property measures being discussed in regional and bilateral free trade agreement negotiations. Reliable quantitative estimates of the Indian role in generic global ARV supply are needed to understand potential impacts of such measures on HIV/AIDS treatment in developing countries.

**Methods:**

We utilized transactional data containing 17,646 donor-funded purchases of ARV tablets made by 115 low- and middle-income countries from 2003 to 2008 to measure market share, purchase trends and prices of Indian-produced generic ARVs compared with those of non-Indian generic and brand ARVs.

**Results:**

Indian generic manufacturers dominate the ARV market, accounting for more than 80% of annual purchase volumes. Among paediatric ARV and adult nucleoside and non-nucleoside reverse transcriptase inhibitor markets, Indian-produced generics accounted for 91% and 89% of 2008 global purchase volumes, respectively. From 2003 to 2008, the number of Indian generic manufactures supplying ARVs increased from four to 10 while the number of Indian-manufactured generic products increased from 14 to 53. Ninety-six of 100 countries purchased Indian generic ARVs in 2008, including high HIV-burden sub-Saharan African countries. Indian-produced generic ARVs used in first-line regimens were consistently and considerably less expensive than non-Indian generic and innovator ARVs. Key ARVs newly recommended by the World Health Organization are three to four times more expensive than older regimens.

**Conclusions:**

Indian generic producers supply the majority of ARVs in developing countries. Future scale up using newly recommended ARVs will likely be hampered until Indian generic producers can provide the dramatic price reductions and improved formulations observed in the past. Rather than agreeing to inappropriate intellectual property obligations through free trade agreements, India and its trade partners - plus international organizations, donors, civil society and pharmaceutical manufacturers - should ensure that there is sufficient policy space for Indian pharmaceutical manufacturers to continue their central role in supplying developing countries with low-priced, quality-assured generic medicines.

## Background

India has emerged as a world leader in generic pharmaceuticals production, supplying 20% of the global market for generic medicines [1]. The emergence of generic sources supplying quality antiretroviral (ARV) medicines at prices much lower than originator prices undoubtedly accelerated the global scale up of HIV/AIDS treatment. From 2002 to 2008, more than 4 million people were started on antiretroviral therapy (ART) in developing countries [2].

To date, the vast majority of people in low- and middle-income countries have been treated with generic ARVs produced by Indian manufacturers unhampered by patent and other intellectual property restrictions [3]. This absence of intellectual property barriers also resulted in the development of improved ARV formulations, such as paediatric dosage forms and fixed-dose combination (FDC) ARVs whereby two or more ARVs are combined into one tablet. As of the end of 2009, the United States Food and Drug Administration and the World Health Organization (WHO) Prequalification Programme approved or pre-qualified 57 adult FDCs and 31 paediatric ARV tablets produced by Indian generic manufacturers but only eight adult FDCs and 14 paediatric ARV tablets produced by non-Indian and originator manufacturers [4-6].

The intellectual property framework that positioned India as the "pharmacy of the developing world", however, is rapidly changing. In 2005, India was obliged to amend its patent law to allow product patents on medicines to comply with the World Trade Organization (WTO) Agreement on Trade Related Aspects of Intellectual Property Rights (TRIPS). The introduction of product patents in India is severely constraining generic competition and supply, particularly for newer medicines. Now, there is a threat that the limited policy space that remains will be further constricted by bilateral or regional free trade agreements. Unfortunately, many free trade agreements that have been concluded or are being negotiated between industrialized and developing countries contain measures that restrict access to medicines [7].

Agreements involving India are of particular concern because of the country's role as a worldwide supplier of low-priced generic medicines. For example, current free trade agreement negotiations between the European Union and India [8,9] include measures that delay or restrict competition from generic medicines, including: patent term extensions beyond the 20 years required by TRIPS; data exclusivity (that could delay the registration of generic medicines); and border enforcement measures that could block international trade in generic medicines when they are suspected of infringing patents in the countries through which they transit. These types of border measures blocked medicines from reaching patients in Africa and Latin America in 2008 and 2009 when European customs authorities seized Indian-produced generics transiting via Amsterdam airport on suspicion that they infringed Dutch patents [10]. All of these measures can delay or restrict competition from generic medicines and are in direct conflict with the 2001 WTO Doha Declaration on TRIPS and Public Health, and medical ethics [8,9].

A better understanding of the role that Indian generic medicines producers play in HIV/AIDS treatment in developing countries will shed light on the potential consequences of recently proposed intellectual property measures for global public health. While their relative importance is widely recognized, reliable quantitative estimates of generic ARVs supplied by Indian producers are not available. The purpose of this paper is to quantify the extent to which Indian pharmaceutical manufacturers have contributed to HIV/AIDS treatment in developing countries to better understand the potential implications of current and future policies that may hamper or restrict market entry of generic ARV manufacturers and generic competition.

## Methods

**Figure 1:**
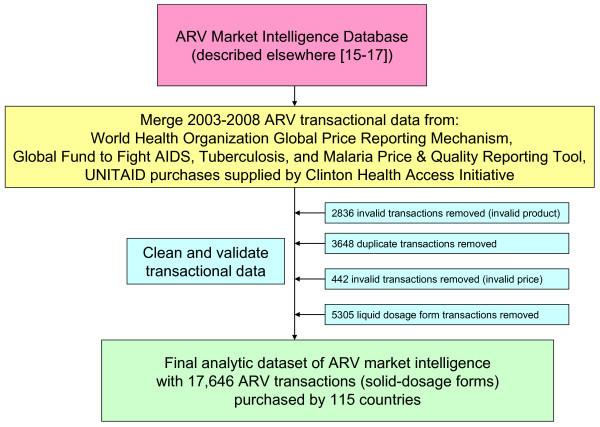
**Description of analytic data set**.

We obtained donor-funded ARV purchase transactions over the 2003-2008 period from the WHO Global Price Reporting Mechanism, the Global Fund to Fight AIDS, Tuberculosis and Malaria's Price & Quality Reporting Tool, and UNITAID as provided by the Clinton Health Access Initiative [11-14]. Antiretroviral transactional data was systematically cleaned and validated using a market intelligence database described elsewhere [15-17]. We excluded transactions for liquid ARV formulations, which resulted in an analytic data set containing 17,646 donor-funded purchases of ARV tablets and capsules made by 115 countries (Figure 1).

Market share by volume is calculated in person-years for Indian generic, non-Indian generic and brand ARVs using WHO-recommended adult doses for persons weighing more than 60 kilogrammes (kg) [18,19]. We provided estimates of producer market share for all ARVs, but also calculated market share among three ARV market niches: paediatric ARVs (all classes), adult nucleoside reverse transcriptase inhibitors (NRTIs) and non-nucleoside reverse transcriptase inhibitors (NNRTIs), and adult protease inhibitors (PIs).

We compared purchase trends for Indian generic, non-Indian generic and brand ARVs, summarizing the number of manufacturers, products/dosage forms, purchases, purchasing countries and value (in US dollars).

We calculated 2008 antiretroviral regimen prices for the most commonly used first-line regimens recommended by the WHO in its 2003 and 2006 treatment guidelines for adults weighing more than 60 kg [18,19]. We expressed regimen prices as price per person per year. Because most ARV price distributions were skewed dramatically by a few high price outliers, we presented regimen prices using median and quartile prices to accurately convey central tendencies. We differentiate regimen composition by using a "+" when multiple tablets are used to create a regimen (e.g., 3TC+NVP+TDF) and a "/" for FDC formulations (e.g., 3TC/NVP/d4T). We plotted 2003-2008 trends in generic ARV regimen prices along with those of innovator ARV regimens offered through differential or tiered prices, as reported to Médecins Sans Frontières (MSF) in its "Untangling the web of ARV price reductions" [20]. We obtained all ARV prices in United States dollars and adjusted them to the January-December 2008 period using the annual US Consumer Price Index [21].

## Results

**Figure 2:**
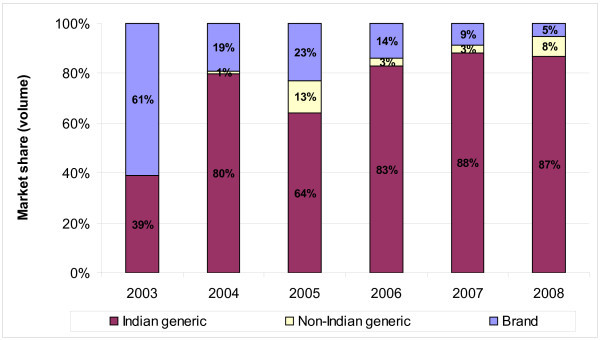
**Overall ARV market share (volume) for Indian generic, non-Indian generic and originator (brand) manufacturers, 2003-2008**.

Our results confirm the prominence of Indian generic manufacturers in the supply of antiretroviral medicines to developing countries. Since 2006, Indian-produced generic ARVs have accounted for more than 80% of the donor-funded developing country market, and comprised 87% of ARV purchase volumes in 2008 (Figure 2).

**Figure 3:**
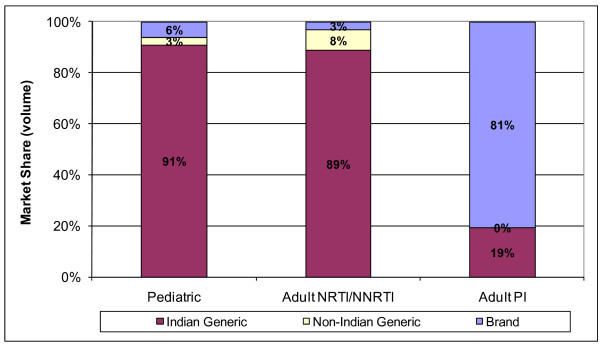
**Adult and paediatric ARV market share (volume) for Indian generic, non-Indian generic and originator (brand) manufacturers, 2008**.

The proportion of ARVs produced by Indian manufacturers is even higher within certain market niches. In 2008, Indian-produced generics accounted for 91% of paediatric ARV volume and 89% of adult NRTI and NNRTI purchases (Figure 3). In contrast, originator companies accounted for the majority (81%) of purchase volumes for adult protease inhibitors (PIs), with Indian generics accounting for only 19%.

The value of the donor-funded, developing country ARV market has exhibited dramatic annual growth over the past several years. By 2008, Indian generic ARVs accounted for 65% of the total value (US$463 million) of ARV purchases reported, while non-Indian generic and innovator ARVs accounted for 13% and 22% of market value, respectively (Table 1). The number of Indian generic manufacturers supplying ARVs to low- and middle-income countries increased from four to 10 from 2003 to 2008, while the number of Indian-produced generic ARV products increased from 14 to 53 over the same period (Table 1).

**Table 1:** Purchase trends for Indian generic, non-Indian generic and originator ARVs, 2003-2008

	2003	2004	2005	2006	2007	2008
**# Countries reporting any ARV purchase**	15	69	86	86	90	100

						

**Indian generic ARVs**						

# manufacturers	4	8	6	7	9	10

# products/dosage forms	14	31	30	37	47	53

# purchases	62	740	1142	1273	2433	5906

# purchasing countries	11	55	70	78	81	96

NRTIs	11	53	66	74	80	92

NNRTIs	6	51	65	63	75	93

PIs	4	17	20	26	31	37

value (USD millions)	0.67	43.84	86.54	93.40	188.08	301.38

						

**Non-Indian generic ARVs**						

# manufacturers	0	2	3	2	3	6

# products/dosage forms	0	5	19	15	18	15

# purchases	0	10	228	124	201	316

# purchasing countries	0	4	10	13	20	29

NRTIs	0	2	9	11	20	25

NNRTIs	0	2	4	3	6	5

PIs	0	0	1	0	0	0

value (USD millions)	0	0.12	27.38	3.72	14.34	58.76

						

**Originator ARVs**						

# manufacturers	6	8	8	7	7	8

# products/dosage forms	18	32	33	39	40	39

# purchases	35	654	1146	976	1284	1116

# purchasing countries	8	50	75	77	79	88

NRTIs	4	40	57	66	63	57

NNRTIs	4	31	52	36	22	14

PIs	4	32	58	67	73	82

value (USD millions)	1.64	29.80	74.39	56.51	83.02	102.62

**Figure 4:**
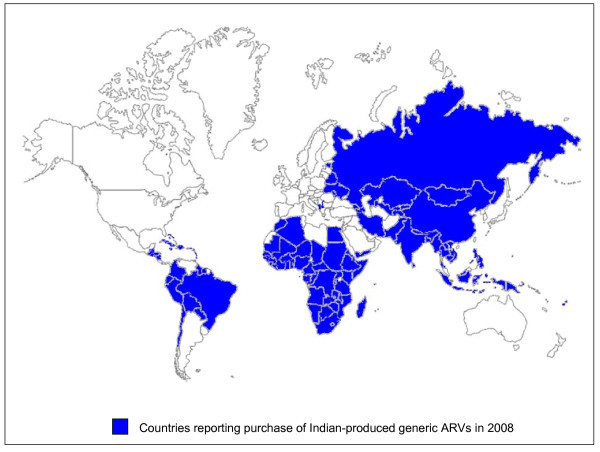
**Countries reporting purchases of Indian generic ARVs in 2008**.

In 2008, 96 of 100 countries reported ARV purchases from Indian generic producers, while only 29 countries reported purchases from non-Indian generic manufacturers (Table 1, Figure 4). Most countries reported purchases of innovator PIs whereas far fewer countries reported generic PI purchases, most likely due to lower prices offered through tiered pricing schemes for brand lopinavir/ritonavir in 2003-2008. The number of countries purchasing Indian-produced generic PIs, however, has steadily increased over the years as global PI volumes have increased and generic pricing has become more competitive with originator tiered prices.

Analysis of Indian-produced generic ARV purchase trends by country reveal India's own reliance on the availability of generic ARVs as demonstrated by nearly 2200 purchases of Indian-produced generic ARVs (Table 2) totalling nearly US$26 million in 2008. Volumes associated with these purchases were sufficient to treat more than 200,000 people with first-line regimens and more than 1000 people with second-line regimens. India reported no purchases for non-Indian generic or innovator ARVs in 2008. Sub-Saharan African countries with high HIV/AIDS disease burdens comprise the remaining top 10 purchasers of Indian-produced generic ARVs (Table 2).

**Table 2:** Summary of Indian-produced generic ARVs for countries with highest 2008 purchase volumes

Purchase volume rank	Country	% of ARV volume supplied by Indian generic producers	Value of Indian- produced generic ARV purchases (USD million)	# Indian-produced generic ARV dosage forms purchased
1	India	100	25.9	14

2	United Republic of Tanzania	96	27.3	13

3	Nigeria	84	27.1	28

4	Ethiopia	96	27.6	24

5	Mozambique	99	15.3	16

6	Zambia	94	20.7	19

7	Namibia	99	15.3	23

8	Democratic Republic of the Congo	99	11.4	25

9	Kenya	82	10.2	14

10	Cameroon	93	15.0	30

**Figure 5:**
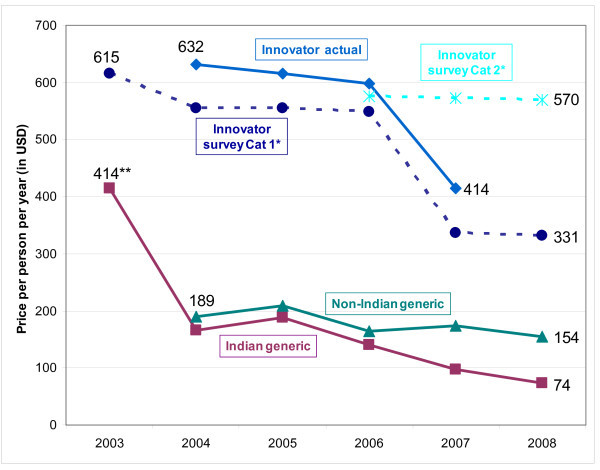
**Price trends for generic 3TC/NVP/d4T30 (fixed-dose combination) and innovator 3TC+NVP+d4T30 (3 individual tablets), 2003-2008**. *Survey prices provided by innovator companies under tiered-pricing [20]. **2003 price is for three individual ARVs (1^st ^FDC purchase reported in 2004).

Robust competition among manufacturers has contributed to substantial price reductions for generic ARVs over the past several years. The most commonly used first-line adult regimen (lamivudine/nevirapine/stavudine30) dropped from $414 per person per year in 2003 to $74 per person per year in 2008 for Indian-produced generics (Figure 5). While regimen prices for non-Indian generic were similar to Indian generic ARVs from 2004 to 2006, by 2008 the non-Indian generic price was two times higher than the Indian generic price. Innovator prices for this first-line regimen, both actual prices contained in our database and survey prices reported to MSF [20], were consistently much higher than generic ARVs across all years. In 2008, innovator regimen prices reported to MSF were 4.5 and 7.7 times higher than Indian generic prices, depending upon the tiered-price category of the purchasing country (Figure 5) [20].

Among many concerns around the future of global ART scale up are higher prices for new WHO-recommended, first-line regimens that utilize zidovudine or tenofovir in place of stavudine [19,22]. As of 2008, the Indian generic global median price for newly recommended tenofovir-based regimens ranged from $246 to $309 per person per year, notably 3.3 to four times higher than the price of the most commonly used older regimen (3TC/NVP/d4T30) (Table 3). Identical regimens, comprised of non-Indian generic and innovator ARVs, are considerably more expensive than the Indian generic versions.

**Table 3:** First-line ARV regimen prices comparisons, 2008

	Indian generic median price (25^th^, 75^th^)	Non-Indian generic median price (25^th^, 75^th^)	Innovator actual median price (25^th^, 75^th^)	Innovator survey price** Cat 1, Cat 2
**First-line regimens from 2003 WHO guidelines:**				

3TC/NVP/d4T30	74 (63, 88)	154* (137, 712)	N/A	331, 570

EFV+3TC/d4T30	131 (126, 193)	229* (196, 656)	N/A	349, 789

3TC/NVP/ZDV	120 (118, 123)	142 (142, 142)	519* (496, 991)	444, 663

EFV+3TC/ZDV	183 (177, 260)	326 (254, 348)	491 (475, 801)	434, 854

				

**New first-line regimens from 2009 WHO guidelines:**				

3TC+NVP+TDF	246 (230, 273)	340 (321, 767)	575 (519, 1254)	490, 867

EFV+3TC+TDF	298 (283, 369)	415 (381, 711)	546 (498, 1064)	508, 1086

FTC/TDF+NVP	257 (247, 301)	387 (386, 537)	641 (569, 1116)	538, 986

EFV+FTC/TDF	309 (300, 397)	461 (446, 480)	612 (548, 926)	556, 1205

N/A insufficient sample size to estimate price

*regimen prices calculated by summing up prices of 3 component ARVs

**Médecins Sans Frontières, "Untangling the web of ARV price reductions" [22]

## Discussion

These analyses quantify and confirm the exceptional role that India has played in providing quality ARVs at low prices to people with HIV/AIDS in developing countries. More than 80% of all donor-funded ARVs purchased since 2006 were supplied by Indian generic manufacturers. Price reductions noted for commonly used historical first-line regimens were a result of robust generic competition among Indian manufacturers in an environment largely void of intellectual property barriers [23,24]. Countries across sub-Saharan Africa with high HIV/AIDS burdens, as well as India, are heavily reliant on the availability of Indian-produced generic ARVs to support their national treatment programmes.

Trade-related and intellectual property-related threats to the supply of generic medicines from India are coming at a time when the prospects of ART scale up are already cloudy. New WHO guidelines recommending early initiation of ART [22] will result in increased numbers of people in need of treatment. At the same time, countries are trying to adopt the new ARV regimens recently recommended by WHO [19,25]. These newer ARVs offer better side-effect and tolerability profiles, but some of the key ARVs are more widely patented and are much more expensive than regimens used in the past. These WHO changes are welcome and help eliminate historical inequities whereby people in resource-poor countries receive a different standard of care than those in rich countries. However, country budgets within the Global Fund to Fight AIDS, Tuberculosis, and Malaria have been cut [26], while pledges and contributions appear flat, raising concerns that funds will not be available in-country to adopt the new WHO recommendations [19,22,25].

## Limitations

Our study captures only donor-funded purchases and not those made by government-funded HIV/AIDS treatment programmes through such countries as Brazil, South Africa and Thailand. Similarly, we had no access to comprehensive and reliable data on patents and other intellectual property barriers and were, therefore, unable to quantitatively examine these issues in our study. While we systematically cleaned and validated all transactional data, we cannot be confident that we have identified all reporting errors in publicly available data. Prices are inconsistently reported to the Global Fund and the WHO Global Price Reporting Mechanism. Whereas some organizations, such as UNITAID and the Supply Chain Management System arm of the United States President's Emergency Plan for AIDS Relief, provide prices for drug costs only, Global Fund-supported countries often report prices that include not only drug costs, but also add-on costs, such as transport, insurance and taxes.

We attribute ARV price reduction primarily to generic competition, but we note that these price decreases were also spurred through the efforts of HIV/AIDS activists, civil society organizations, national governments, foundations and other international organizations.

Despite these limitations, our research provides valuable quantitative information demonstrating the critical role that Indian generic pharmaceutical manufacturers play in the global treatment of HIV/AIDS in developing countries. These results can and should be used in ongoing and future discussions around intellectual property and access to medicines.

## Conclusions

Free trade agreements that may create new intellectual property obligations for India can increase ARV prices, impede the development of acceptable dosage forms, and delay access to newer and better ARVs. Such measures can undermine the international goal to achieve universal access to HIV/AIDS interventions and the 2001 WTO Doha Declaration on TRIPS and Public Health [25]. Rather than agreeing to inappropriate intellectual property obligations, India and its trade partners - along with international organizations, donors, national governments, civil society and pharmaceutical manufacturers - should ensure that there is sufficient policy space for the Indian generic industry to continue its central role in supplying developing countries with low-cost, quality-assured generic medicines.

## Competing interests

The authors declare that they have no competing interests.

## Authors' contributions

BW designed and coordinated the study, participated in data cleaning and data analysis, and was the lead author on this paper. ED performed data cleaning and data analysis. SM contributed to data analysis, writing of the manuscript, and editing for important content. All authors read and approved the final version of the manuscript.

## References

[B1] Perlitz U India's pharmacuetical industry on course for globalisation. Frankfurt 2008

[B2] World Health Organization, UNAIDS, UNICEF Towards universal access: Scaling up priority HIV/AIDS interventions in the health sector. Progress report 2009. Geneva 2009

[B3] Médecins Sans Frontières Untangling the web of antiretroviral price reductions http://utw.msfaccess.org/

[B4] World Health Organization Health Systems and Services: Prequalification of Medicines Programme http://apps.who.int/prequal/

[B5] President's Emergency Plan for AIDS Relief Approved and Tentatively Approved Antiretrovirals in Association with the President's Emergency Plan

[B6] United States Food and Drug Administration Drugs @ FDA: FDA Approved Drug Products http://www.accessdata.fda.gov/scripts/cder/drugsatfda/index.cfm

[B7] Correa C Implications of bilateral free trade agreements on access to medicines Bull World Health Organ 2006 13 5 399 404 10.2471/BLT.05.023432 16710551 PMC2627342

[B8] Von Schoen-Angerer T Letter to Karel de Gucht, European Commissioner for Trade http://www.msfaccess.org/fileadmin/user_upload/medinnov_accesspatents/MSF%20letter%20to%20Trade%20Commissioner%20April%202010.pdf

[B9] De Gucht K Letter to Tido von-Schoen-Angerer, Executive Director, MSF Campaign for Access to Essential Medicines

[B10] Abbott F Seizure of generic pharmaceuticals in transit based on allegations of patent infringement: a threat to international trade, development and public welfare World Intellectual Property Organization Journal 2009 13 43

[B11] UNITAID http://www.unitaid.eu/

[B12] William J Clinton Foundation: Treating HIV/AIDS and malaria http://www.clintonfoundation.org/what-we-do/clinton-health-access-initiative

[B13] World Health Organization Global price reporting mechanism http://www.who.int/hiv/amds/gprm/en/

[B14] Global Fund to Fight AIDS, Tuberculosis and Malaria Price & Quality Reporting http://pqr.theglobalfund.org/PQRWeb/Screens/PQRLogin.aspx?Lang=en-GB

[B15] Waning B Kaplan W King A Lawrence D Leufkens H Fox M Global strategies to reduce the price of antiretroviral medicines: evidence from transactional databases Bull World Health Organ 2009 13 7 520 528 http://www.who.int/bulletin/volumes/87/7/08-058925.pdf 10.2471/BLT.08.058925 19649366 PMC2704041

[B16] Waning B Kaplan W Fox M Boyd-Boffa M King A Lawrence D Soucy L Mahajan S Leufkens H Gokhale M Temporal trends in generic and brand prices of antiretroviral medicines procured with donor funds in developing countries J Gen Med 2010 13 159 175 10.1057/jgm.2010.6

[B17] Waning B Kyle M Diedrichsen E Soucy L Hochstadt J Bärnighausen T Moon S Intervening in global markets to improve access to HIV/AIDS treatment: an analysis of international policies and the dynamics of global antiretroviral medicines markets Glob & Health 2010 13 9 http://www.globalizationandhealth.com/content/6/1/9 10.1186/1744-8603-6-9 PMC2883977 20500827

[B18] World Health Organization Scaling up antiretroviral therapy in resource-limited settings: treatment guidelines for a public health approach, 2003 revision. Geneva 2004

[B19] World Health Organization Antiretroviral therapy for HIV infection in adults and adolescents in resource-limited settings: towards universal access. Geneva 2006

[B20] Médecins Sans Frontières Untangling the web of antiretroviral price reductions 2003 4-11 Geneva: Médecins sans Frontières

[B21] International Monetary Fund World Economic Outlook Database. Washington DC 2010

[B22] World Health Organization Rapid advice: antiretroviral therapy for HIV infection in adults and adolescents. Geneva 2009 23741771

[B23] Ford N Wilson D Chaves GC Lotrowska M Kijtiwatchakul K Sustaining access to antiretroviral therapy in the less-developed world: lessons from Brazil and Thailand AIDS 2007 13 S21 S29 10.1097/01.aids.0000279703.78685.a6 17620749

[B24] 't Hoen E The Global Politics of Pharmaceutical Monopoly Power: Drug Patents, Access, Innovation and the Application of the WTO Doha Declaration on TRIPS and Public Health 2009 Diemen: AMB Publishers

[B25] World Health Organization Prioritizing second-line antiretroviral drugs for adults and adolescents: a public health approach. Geneva 2007

[B26] Paton W Communication to all CCMs and PRs on 10% savings on round 8 2008 Geneva: Global Fund to Fight AIDS, Tuberculosis and Malaria

